# Spatial Encoding of Translational Optic Flow in Planar Scenes by Elementary Motion Detector Arrays

**DOI:** 10.1038/s41598-018-24162-z

**Published:** 2018-04-11

**Authors:** Julien Lecoeur, Emily Baird, Dario Floreano

**Affiliations:** 1 Laboratory of Intelligent Systems, School of Engineering, École Polytechnique Fédérale de Lausanne, Lausanne, CH-1015 Switzerland; 2 Department of Biology, Lund University, Lund, Sweden

## Abstract

Elementary Motion Detectors (EMD) are well-established models of visual motion estimation in insects. The response of EMDs are tuned to specific temporal and spatial frequencies of the input stimuli, which matches the behavioural response of insects to wide-field image rotation, called the optomotor response. However, other behaviours, such as speed and position control, cannot be fully accounted for by EMDs because these behaviours are largely unaffected by image properties and appear to be controlled by the ratio between the flight speed and the distance to an object, defined here as relative nearness. We present a method that resolves this inconsistency by extracting an unambiguous estimate of relative nearness from the output of an EMD array. Our method is suitable for estimation of relative nearness in planar scenes such as when flying above the ground or beside large flat objects. We demonstrate closed loop control of the lateral position and forward velocity of a simulated agent flying in a corridor. This finding may explain how insects can measure relative nearness and control their flight despite the frequency tuning of EMDs. Our method also provides engineers with a relative nearness estimation technique that benefits from the low computational cost of EMDs.

## Introduction

Flying insects like flies, bees, moths and dragonflies are well known for their exquisite flight control capabilities. Despite their tiny brains and relatively crude visual systems, they routinely fly through cluttered environments, navigating over large distances and deftly avoiding obstacles in their path. To control their flight, insects use optic flow, defined as the pattern of apparent motion generated on their retina as they move through a scene^1^. Granted sufficient image texture, optic flow measures the apparent angular velocity of surrounding objects. For purely translational motion, translational optic flow (*TOF*) becomes proportional to the relative nearness — noted *η* — defined here as the ratio between the flight speed and the distance to an object^2^. Many complex behaviours exhibited by flying insects, such as visual odometry, landing, position, speed and height control are regulated using information extracted from optic flow (for reviews see^3–5^). Similar optic flow based strategies have also been successfully used to generate autonomous behaviour in miniature flying robots^6–11^, and even bio-hybrid robots^12^. Optic flow based strategies are interesting for the development of control systems in miniature flying vehicles because they have low computational cost and can be implemented on small platforms where constraints in weight and computational power are important.

The Elementary Motion Detector (EMD), introduced by Hassenstein and Reichardt^13,14^, is a well-established biological model for visual motion estimation. The model was originally developed to account for the turning responses made by walking beetles – known as the optomotor response – when presented with wide field yaw image motion^13^ and has since been shown to match the optomotor responses of a wide range of insects^15^. The EMD performs spatio-temporal correlation of the signals from two adjacent photoreceptors and requires only two low-pass filters, two subtractions and one multiplication to provide an estimate of visual motion. This organisation is thought to exist in the early processing stages of the insect visual system in the form of hundreds of EMD units, each taking input from neighbouring photoreceptors around the panoramic field of view of insect eyes.

Neurophysiological studies^16–21^ have provided good evidence for the EMD as a candidate model for motion detection in insect brains, although recent literature shows evidence for both Barlow-Levick^22^ and Hassenstein-Reichardt models^13,14^, suggesting a hybrid implementation (for reviews see^15,23^). Indeed, models integrating the output of EMD arrays from a wide field of view – mimicking the tangential cells in the lobula plate of flies^18^ – have been shown to detect the direction and amplitude of ego-rotations^9,24^, and to perform control of translational motion with simulated agents^25–29^ and robotic agents^8,30–32^.

One of the key features of the EMD model is its dependency on the spatial structure of the scene^33–37^. Both the angular image speed tuning and the temporal frequency tuning of the EMD form a bell shape, with a maximum response at a frequency defined by its characteristics – namely, its integration time and interommatidial angle. While the frequency tuning of the EMD model mimics that observed in the optomotor response to rotational motion, strong support for the model as a basis for translational motion detection is lacking. Behavioural experiments suggest that insects are able to use translational optic flow to correctly estimate relative nearness independently of the spatial structure of the visual input^3,38–40^. This is something that cannot be derived unambiguously from the raw EMD signals because of its bell-shaped tuning to angular speed that is not a monotonic function. Limitations in EMD-based control of translational motion due to the drop in EMD response at low distance from a surface, causing collisions into the surface, have also been reported^28,29^.

Here, we present a novel approach that suggests how the output of EMD arrays could indeed provide the basis for translational motion control in both insects and robotic agents. We show that, although the response of a single EMD does not provide a reliable measurement of angular image speed, comparing responses across an array of EMDs can provide an unambiguous estimate of relative nearness. We study analytically the response of an azimuthally distributed array of EMDs when moving along an planar surface covered by a pattern with a natural distribution of spatial frequencies^41–44^. This surface models either large objects on the sides of the agent, or the ground bellow the agent. We show that, when the ratio between the speed of the agent and its distance to the surface is higher than a threshold we call *η*_min_, the angular location of the EMD with maximum response provides an unambiguous estimate of relative nearness. Our estimator performs best at low distance from the surface – in cases where the raw EMD output provides ambiguous estimates of relative nearness. We then discuss how this finding could be used for flight control, and how the model parameters could be dynamically adapted to enhance the relative nearness estimation. Finally, the proposed EMD-based relative nearness estimator is validated in closed-loop control of a simulated agent.

## Model

**Figure 1:**
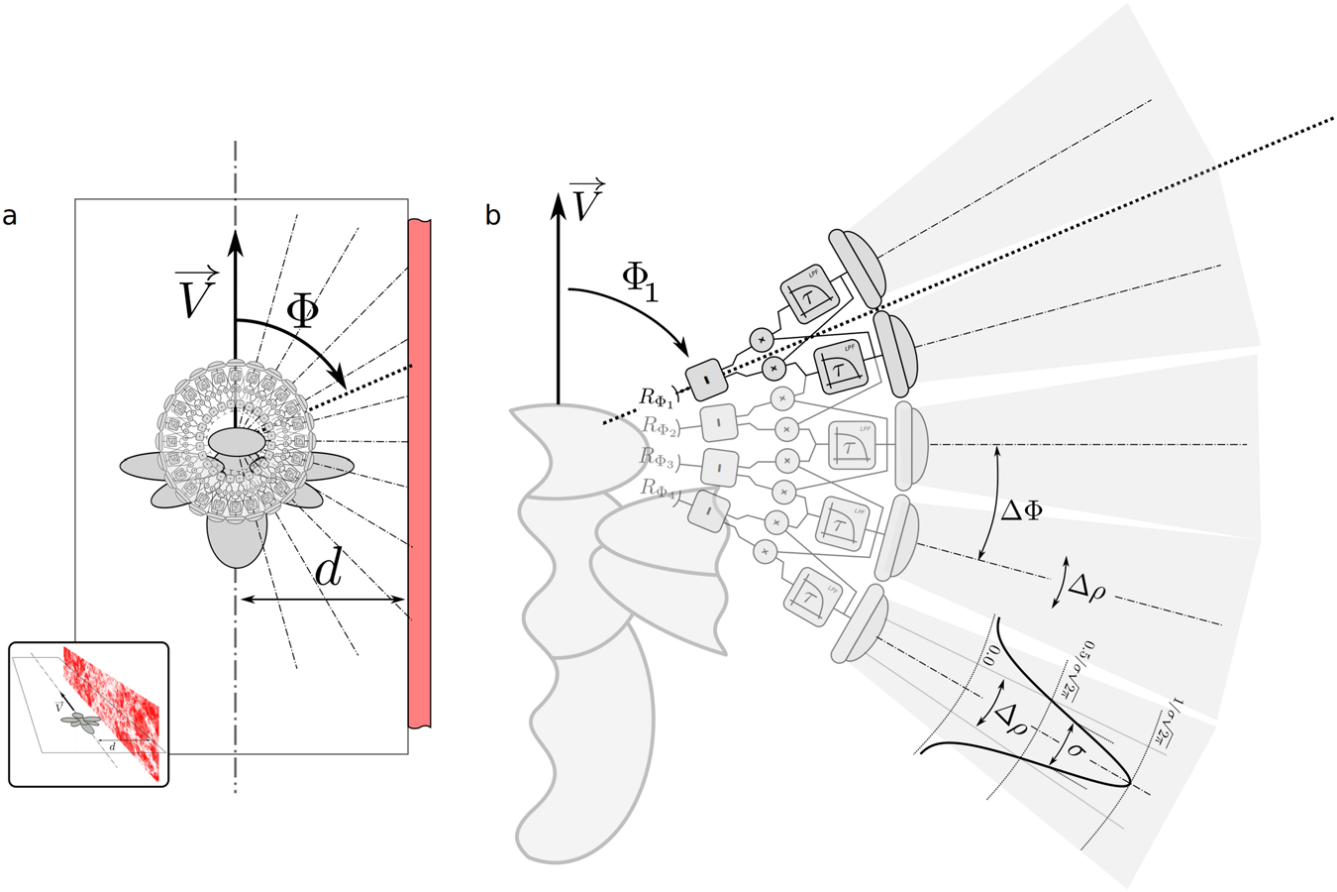
Geometry of the Model. (**a**) Top view of the model. The flying agent — here represented as a bee — moves along a linear trajectory shown as a mixed dashed line. It flies at a speed *V* and at distance *d* from a flat surface, which is covered with a pattern that represents natural spatial frequencies. The agent sees the surface on its right. Viewing directions are defined by the angle Φ, with Φ = 0° for the frontal viewing direction, Φ = 90° for the viewing direction pointing to the right, and Φ = 180° for the backward viewing direction. Overlaid on top of the agent is represented the array of photoreceptors and the array EMD networks considered in this study. The photoreceptors are aligned on a plane that is orthogonal to the patterned surface. Inset: Perspective view of the model. (**b**) Model of the eye and array of EMDs. The eye of the agent is composed of a planar array of independent photoreceptors here represented by five lens-like units. The network of EMDs is retinoptically organized with each EMD taking input from two consecutive photoreceptors. Each one of the four EMDs represented here is composed of two temporal low-pass filter blocks (square blocks labeled *τ*), two multiplication blocks (circular blocks labeled×) and one subtraction block (square blocks labeled −).

Let us consider an agent — be it biological or artificial — flying in an environment composed of a flat surface (Fig. 1a). This surface could represent the ground below a flying agent, or one of the two vertical walls of the corridors commonly used for behavioural studies of insects and birds (for example^3,40,45–48^).

The flying agent moves at speed *V* and distance *d* to the surface (Fig. 1a). Let us define the azimuth angle Φ as the angle between the front of the agent and a viewing direction. We will refer to the viewing direction Φ = 90° as the “lateral region” of the field of view, but this could equally be the “ventral region” of the field of view if the surface was below the agent.

In order to mimic the properties of a real world environment, the flat surface is covered with a pattern that contains a natural distribution of spatial frequencies^41–44,49^, i.e. its power spectrum follows a distribution of frequencies in 1/*f*  ^2^ (Fig. 1-inset).

The eye of the flying agent is composed of a planar array of photoreceptors (Fig. 1b). This plane is orthogonal to the patterned surface and it contains the agent velocity vector $\overrightarrow{V}$. Each photoreceptor points to a different azimuth angle Φ and has an acceptance angle Δ*ρ*. Consecutive photoreceptors are separated by an angle ΔΦ. The receptivity function of an photoreceptor is approximated by a Gaussian window centered on Φ with standard deviation *σ* as in previous studies^26,27,50–52^. The acceptance angle of a photoreceptor — noted Δ*ρ* — is defined as the full width at half maximum of the Gaussian window^53^.

A series of EMDs^14,34,35^ takes input from the photoreceptor array. The output ${R}_{{{\rm{\Phi }}}_{i}}$ of the EMD circuit pointed at the direction Φ_*i*_ is given by the difference of the results of two multiplications (Fig. 1b). The first multiplication is the product of the low-pass filtered signal of the photoreceptor pointed at ${{\rm{\Phi }}}_{i}-\frac{{\rm{\Delta }}{\rm{\Phi }}}{2}$ and the unfiltered signal of the photoreceptor pointed at ${{\rm{\Phi }}}_{i}+\frac{{\rm{\Delta }}{\rm{\Phi }}}{2}$. The second multiplication is the product of the unfiltered signal from the photoreceptor pointed at ${{\rm{\Phi }}}_{i}-\frac{{\rm{\Delta }}{\rm{\Phi }}}{2}$ and the low-pass filtered signal of the photoreceptor pointed at ${{\rm{\Phi }}}_{i}+\frac{{\rm{\Delta }}{\rm{\Phi }}}{2}$.

### Predicted Steady-state EMD Response

In this section we derive the expression of the EMD output value *R* as a function of five parameters: the azimuth angle Φ, the agent speed *V*, the distance between the agent and the surface *d*, the inter-ommatidial angle ΔΦ, and the time constant *τ* of the EMD low-pass filter blocks.

The EMD used in this study is a balanced correlator^14^ composed of two linear low pass filters, one multiplication and one subtraction. The mean EMD response to a moving broadband image can be expressed as the sum of its responses to the individual sinusoidal components of the image, weighted by the power spectrum of the image^37^. For a pattern containing a naturalistic distribution of frequencies – i.e. a power spectrum in 1/*f  *^2^ – the mean EMD response is thus given in equation (1).

1 $${R}_{{\rm{\Phi }}}={\int }_{{f}_{min}}^{{f}_{max}}\,\frac{1}{{f}^{2}}{R}_{{\rm{\Phi }}}^{f}\,df$$

where ${R}_{{\rm{\Phi }}}^{f}$ is the response of the EMD pointed at the viewing direction Φ for a surface covered with a pattern that contains only one spatial frequency *f*, i.e. a sinusoidal grating. In equation (1) the integral computes summation across a range of frequencies. Note that this does not imply that frequency summation is implemented in insect nervous system and thus does not require additional neural computation. The frequency summation is however needed in this study to predict the EMD response to a signal that is itself the sum of sinusoidal components of varying frequencies.

The response *R*_*sin*_ of one EMD to a sinusoidal stimulus was derived in a previous study^35^ for the case of a rotating drum patterned on its inner surface, and is shown in equation (2).

2 $${R}_{sin}={\rm{\Delta }}{I}^{2}\,\sin \,(2\pi \frac{{\rm{\Delta }}{\rm{\Phi }}}{\lambda })\frac{\tau \omega }{1+{\tau }^{2}{\omega }^{2}}$$

where Δ*I* is the amplitude of the sinusoidal stimulus, *ω* is the frequency of the stimulus, *λ* is its angular period, ΔΦ is the inter-ommatidial angle, and *τ* is the time constant of the low pass filter.

While *R*_*sin*_ was derived with the assumption that Δ*I*, *λ* and *ω* were constant across the field of view^35^, in our case (Fig. 1a) they vary depending on the azimuth angle as well as on the position and speed of the agent. Let us introduce the apparent signal amplitude $\widehat{{\rm{\Delta }}I}={\rm{\Delta }}I(f,{\rm{\Delta }}{\rm{\Phi }},{\rm{\Phi }},d)$, the apparent angular period $\widehat{\lambda }=\lambda (f,{\rm{\Phi }},d)$, and the apparent angular frequency $\widehat{\omega }=\omega (f,V)$. For example, the apparent angular period will decrease for increasing distance to the wall, the apparent angular period will also be maximum for Φ = 90° and tend to 0 for Φ → 0° and Φ → 180°. We can thus reformulate equation (2) for our case as equation (3). The expressions of $\widehat{{\rm{\Delta }}I}$, $\widehat{\lambda }$, and $\widehat{\omega }$ are shown in equation (4), for more details see Supplementary Section S2.

3 $${R}_{{\rm{\Phi }}}^{f}={\widehat{{\rm{\Delta }}I}}^{2}\,\sin \,(2\pi \frac{{\rm{\Delta }}{\rm{\Phi }}}{\widehat{\lambda }})\frac{\tau \widehat{\omega }}{1+{\tau }^{2}{\widehat{\omega }}^{2}}$$

4 $$\{\begin{array}{ccc}\hat{\omega } & = & 2\pi \frac{V}{{\rm{\Lambda }}}\\ \hat{{\rm{\Delta }}I} & \approx  & \exp \,(-2{\pi }^{2}\frac{{(0.45{\rm{\Delta }}{\rm{\Phi }})}^{2}}{{\lambda }^{2}})\\ \hat{\lambda } & = & \arctan (\tan (\frac{\pi }{2}-{\rm{\Phi }})+\frac{{\rm{\Lambda }}}{2d})-\arctan (\tan (\frac{\pi }{2}-{\rm{\Phi }})-\frac{{\rm{\Lambda }}}{2d})\end{array}$$

By substituting equation (4) in equation (3), we obtain equation (5).

5 $${R}_{{\rm{\Phi }}}^{f}={\textstyle \tfrac{2\tau \pi Vf}{1+{\tau }^{2}{(2\pi Vf)}^{2}}}.\exp ({\textstyle \tfrac{-2{\pi }^{2}{(0.45{\rm{\Delta }}{\rm{\Phi }})}^{2}}{{(A-B)}^{2}}}).\,\sin ({\textstyle \tfrac{2\pi {\rm{\Delta }}{\rm{\Phi }}}{A-B}}),\,{\rm{w}}{\rm{h}}{\rm{e}}{\rm{r}}{\rm{e}}\,\{\begin{array}{ccc}A & = & \arctan (\tan ({\textstyle \tfrac{\pi }{2}}-{\rm{\Phi }})+{\textstyle \tfrac{2\pi Vf}{2d}})\\ B & = & \arctan (\tan ({\textstyle \tfrac{\pi }{2}}-{\rm{\Phi }})-{\textstyle \tfrac{2\pi Vf}{2d}})\end{array}$$

The complete EMD output given by the integral in equations (1) and (5) is approximated as a discrete sum by considering a finite number ${N}_{f}\gg 1$ of spatial frequencies *f*_*k*_, as shown in equation (6).

6 $$\begin{array}{l}{R}_{{\rm{\Phi }}}\approx \sum _{k=0}^{{N}_{f}-1}\,\tfrac{1}{{f}_{k}^{2}}{R}_{{\rm{\Phi }}}^{{f}_{k}},\,{\rm{where}}\,{f}_{k}={f}_{{\min }}+k.\tfrac{{f}_{{\max }}-{f}_{{\min }}}{{N}_{f}-1}\end{array}$$

The range of spatial frequencies *f*_min_ and *f*_max_ (see Supplementary Section S1) was chosen so that they do not interfere with the results of the study. The maximum spatial period is several orders of magnitude larger than the length covered by the agent flying at the maximum speed during an EMD integration time. The minimum spatial period is small compared to the length covered by one acceptance angle *ρ* at the smallest considered distance to the wall, and is thus filtered by the gaussian acceptance angle convolution, which also avoids potential issues of spatial aliasing^54^.

## Theoretical Results

In this section, we analyse theoretical predictions of the response of an EMD array to an planar surface covered with a natural pattern. We evaluated equation (6) for varying values of the five parameters Φ, *V*, *d*, ΔΦ and *τ* (see Supplementary Table S1). These results are analysed in the following paragraphs.

We first show that the value of the EMD output is not a reliable estimation of relative nearness (i.e. *V*/*d*) in that a single value of EMD output can not be unambiguously associated to a single value of the *V*/*d* ratio. Then, we introduce the angle Ψ, which is obtained from the azimuthal location of maximum output in the EMD array. We show that the angle Ψ covaries monotonically – though non-linearly – with *V*/*d*, and thus provides an unambiguous estimate of relative nearness.

### EMD Response Across the Visual Field

When a flying agent is moving in straight line at constant speed, in a purely translational motion (Fig. 1a), translational optic flow is proportional to flight speed *V* and inversely proportional to the distance to an object in the scene^2,55^: ${\rm{TOF}}({\rm{\Phi }})=\frac{V}{{D}_{{\rm{\Phi }}}}\,\sin ({\rm{\Phi }})$. For the planar surface shown in Fig. 1, which is aligned with the velocity vector and at a distance *d* from the agent, the distance to the surface in the viewing direction Φ is *D*_Φ_ = *d*/sin(Φ). Translational optic flow can then be obtained geometrically with equation (7) which is positively correlated to flight speed *V* and inversely correlated to distance *d*. Note that translational optic flow at viewing angle Φ = 90° yields a maximum value — noted *TOF*_90_ — that is equal to the relative nearness *η* = *V*/*d*.

7 $$TOF({\rm{\Phi }})=\frac{V}{d}\,{\sin }^{2}\,({\rm{\Phi }})$$

**Figure 2:**
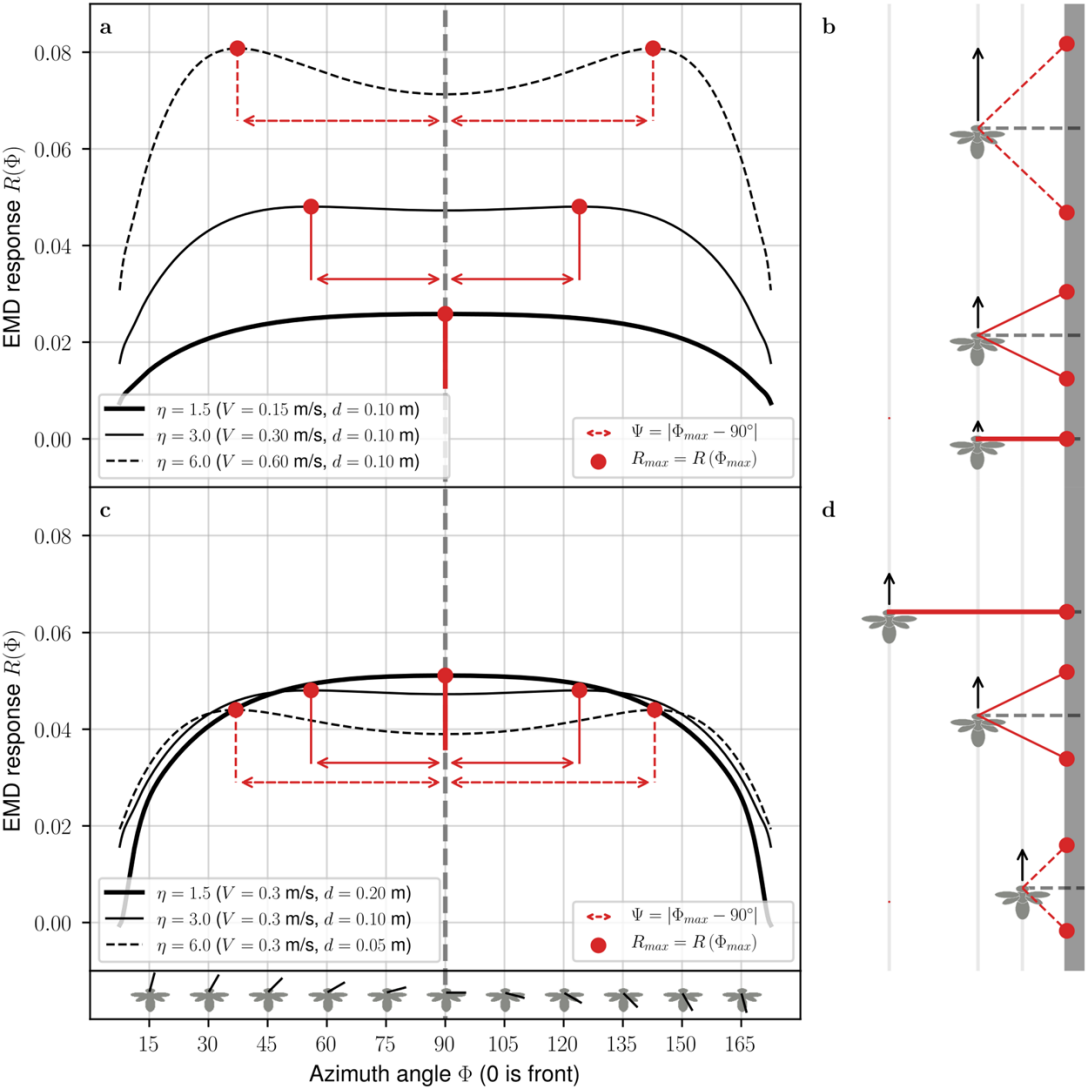
Distribution of EMD output *R* across the visual field for varying speed and varying distance. (**a**,**c**) The EMD output *R* is shown as a function of the azimuth angle Φ. Each black curve represents *R* for a specific value of *V* and *d*. The red dots represent, for each curve, the maximum EMD output across the visual field. The azimuth angle where *R* is maximum is noted Φ_max_ and the maximum value of *R* is noted *R*_max_. The red arrows represent the angle Ψ (defined as Ψ = |Φ_max_ − 90°|) i.e. the angular deviation of the maximum EMD response from the side of the field of view (Φ = 90°). For both graphs, the inter-ommatidial angle and time constant of the low pass filter are kept constant at ΔΦ = 3° and *τ* = 10 ms. (**a**,**b**) The distance to the surface *d* is kept constant at 10 cm for flight speeds 0.15 m/s, 0.30 m/s and 0.60 m/s. (**c**,**d**) The flight speed *V* is kept constant at 30 cm/s, for distances to surface of 5 cm, 10 cm and 20 cm. (**b**,**d**) Schematic representation of the agent flying alongside the vertical surface for the different values of *V* and *d*. The location of maximum EMD response is represented by red dots at the location they would project on the patterned surface. The angle Ψ is equal to 0 for the lower value of the ratio *V*/*d* (thick solid lines), and it increases with increasing *V*/*d* ratios (solid lines and dashed lines).

For a planar EMD array, the absolute value of the EMD response *R* increases at all azimuth angles with increasing flight speed in the range of flight speeds considered in our study (Fig. 2a). At higher flight speeds, the EMD response reaches a maximum then decreases with increasing flight speed (see Supplementary Fig. S3). However, *R* does not always increase with decreasing distance to the surface (Fig. 2c), contrary to optic flow which increases with decreasing distance. For example, with the EMD parameters used for Fig. 2, *R* increases for decreasing values of *d* only in the extreme frontal and rear parts of the field of view (in the ranges Φ ∈ [0°, 30°] and Φ ∈ [150°, 180°]). In most of the field of view (Φ ∈ [45°, 135°]), *R* increases with increasing values of *d*, which is the opposite of a relative nearness estimator.

**Figure 3:**
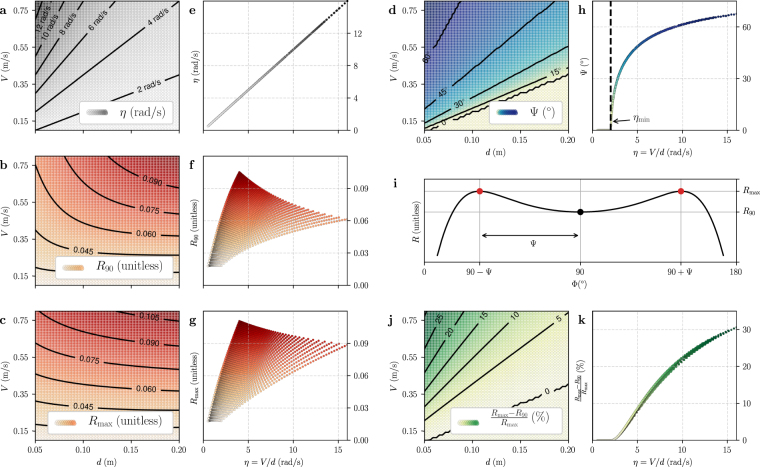
Comparison of raw EMD response and Ψ angle as estimators of relative nearness. (**a**,**e**) Relative nearness, computed geometrically as *η* = *V*/*d*. The unit is rad/s because this is equivalent to the angular image speed. (**b**,**f**) The EMD response at 90° is defined as *R*_90_ = *R*_(Φ=90°)_. (**c**,**g**) The maximum EMD response is defined as ${R}_{{\rm{\max }}}={R}_{({\rm{\Phi }}={{\rm{\Phi }}}_{{\rm{\max }}})}$. (**d**,**h**) Deviation of the location of maximum EMD response Ψ = |Φ_max_ − 90°|. Left (**a–d**) Values given as functions of flight speed *V* and distance *d*. Right (**e–h**) Values given as functions of the relative nearness *η* which is equivalent to the translational optic flow at viewing angle 90 degrees *η* = *TOF*_90_ = *V*/*d*. In all plots, the inter-ommatidial angle and time constant of the low pass filter are kept constant at ΔΦ = 3° and *τ* = 10 ms. (**i**) Graphical representation of *R*_90_, *R*_max_ and Ψ on EMD response *R* shown as function of viewing angle Φ. (**j**,**k**) Relative difference between *R*_max_ and *R*_90_ (given in percents), it indicates the maximum level of noise allowing the two maxima to be detected.

Let us define *R*_90_ as the EMD response at Φ = 90°, and *R*_max_ as the maximum EMD response which is located at Φ = Φ_max_ (Fig. 2a). Neither *R*_90_ or *R*_max_ provide a correct estimate of relative nearness. While they both depend on flight speed and distance to the surface (Fig. 3b,c), the isocurves of *R*_90_ and *R*_max_ are not at a constant *V*/*d* ratio, as is the case for relative nearness (Fig. 3a). This means that, unlike relative nearness, a single *V*/*d* ratio can correspond to different *R*_90_ or *R*_max_ values. An agent flying at speed *V* and distance *d* to the surface should measure the same relative nearness when flying at double speed and double distance because the ratio *V*/*d* is the same in both cases. However this is not the case for *R*_90_ and *R*_max_ which yield two different values when the agent is flying at speed *V* at distance *d*, and at speed 2*V* at distance 2*d*.

Conversely, a single value of *R*_90_ or *R*_max_ can correspond to different *V*/*d* ratios. The ambiguity of the estimate provided by *R*_90_ and *R*_max_ is clearly visible when they are displayed as function of *V*/*d*, i.e. the relative nearness (Fig. 3f,g). A single value of *R*_90_ or *R*_max_ can correspond to a wide range of relative nearness. For instance, for *R*_90_ = 0.06 on Fig. 3f, the relative nearness can be anywhere between 2 rad.s^−1^ and 16 rad.s^−1^. Similarly, for *R*_max_ = 0.06 on Fig. 3g, the relative nearness can be anywhere between 2 rad.s^−1^ and 10 rad.s^−1^.

### Deviation of Maximum EMD Response Ψ as Estimation of Relative Nearness

It is interesting to note that the maximum EMD response (noted *R*_max_ and indicated by red dots in Fig. 2) is not always located where the translational optic flow (defined in equation 7 as the image angular velocity) is the highest, ie. at Φ = 90°. The location of the maximum EMD response (noted Φ_max_) is thus not equivalent to the location of the maximum translational optic flow. Let us define Ψ, the deviation of the maximum EMD response from the side of the field of view as

8 $${\rm{\Psi }}=|{{\rm{\Phi }}}_{{\rm{\max }}}-90^\circ |$$

The fact that EMD response is not highest at Φ = 90° can be explained by two facts. First, the bell-shaped speed tuning of EMDs when presented to broadband images^37^ has a maximum at a specific angular speed (see Supplementary Fig. S3). Second, the apparent image speed is lower in the frontal and rear parts of the visual field than at Φ = 90° as shown in equation (7). Thus, at high relative nearness, the EMD may respond with a larger value to the lower angular image speed at Φ = 90 ± Ψ, than to the larger angular image speed at Φ = 90°.

With fixed distance to the surface, Ψ increases with increasing flight speed (Fig. 2a,b). With fixed flight speed, Ψ increases with decreasing distance (Fig. 2c,d). Thus, Ψ is increasing for increasing values of the ratio *V*/*d*, which is the relative nearness. As a consequence, we propose to use Ψ — rather than *R* — to estimate relative nearness.

Contrary to *R*_90_ and *R*_max_, the isocurves of Ψ are at a constant *V*/*d* ratio (Fig. 3d), which is also the case for the relative nearness (Fig. 3a). Moreover a single value of Ψ corresponds to a single *V*/*d* ratio (Fig. 3h), like relative nearness (Fig. 3e).

The function $\eta \mapsto {\rm{\Psi }}$ is monotonically increasing (Fig. 3h). However this function is not strictly increasing for the lower values of *η* where Ψ = 0° (left region of Fig. 3h). This means that Ψ can be used to compare relative nearness in different regions of the field of view (as described later in the experimental section) only when relative nearness is higher than a threshold value.

### Threshold for an Unambiguous Estimation of Relative Nearness

The deviation of maximum EMD response Ψ is equal to zero (i.e. Φ_max_ = 90°) for all values of *η* below a threshold *η*_min_ (lower right corner of Fig. 3d and left region of Fig. 3h). If *η* < *η*_min_, then Ψ is null and provides no useful information on relative nearness. However if *η* > *η*_min_, then Ψ is greater than zero and can be used to estimate relative nearness. In other words, the agent needs to fly sufficiently fast and/or close to the surface to get a relative nearness estimate from Ψ.

For Ψ to be measureable in a practical implementation, the maxima of the EMD response have to be sufficiently separated (Fig. 3i). The higher the relative nearness, the easier it is to detect the maxima, as shown by the relative difference between the maximum EMD response *R*_max_ and the EMD response between the maxima *R*_90_ (Fig. 3j). For example, for a relative nearness of *η* = 5 rad.s^−1^, our model predicts approximately 8% difference between *R*_max_ and *R*_90_. This value increases to approximately 22% for *η* = 10 rad.s^−1^ (Fig. 3k).

**Figure 4:**
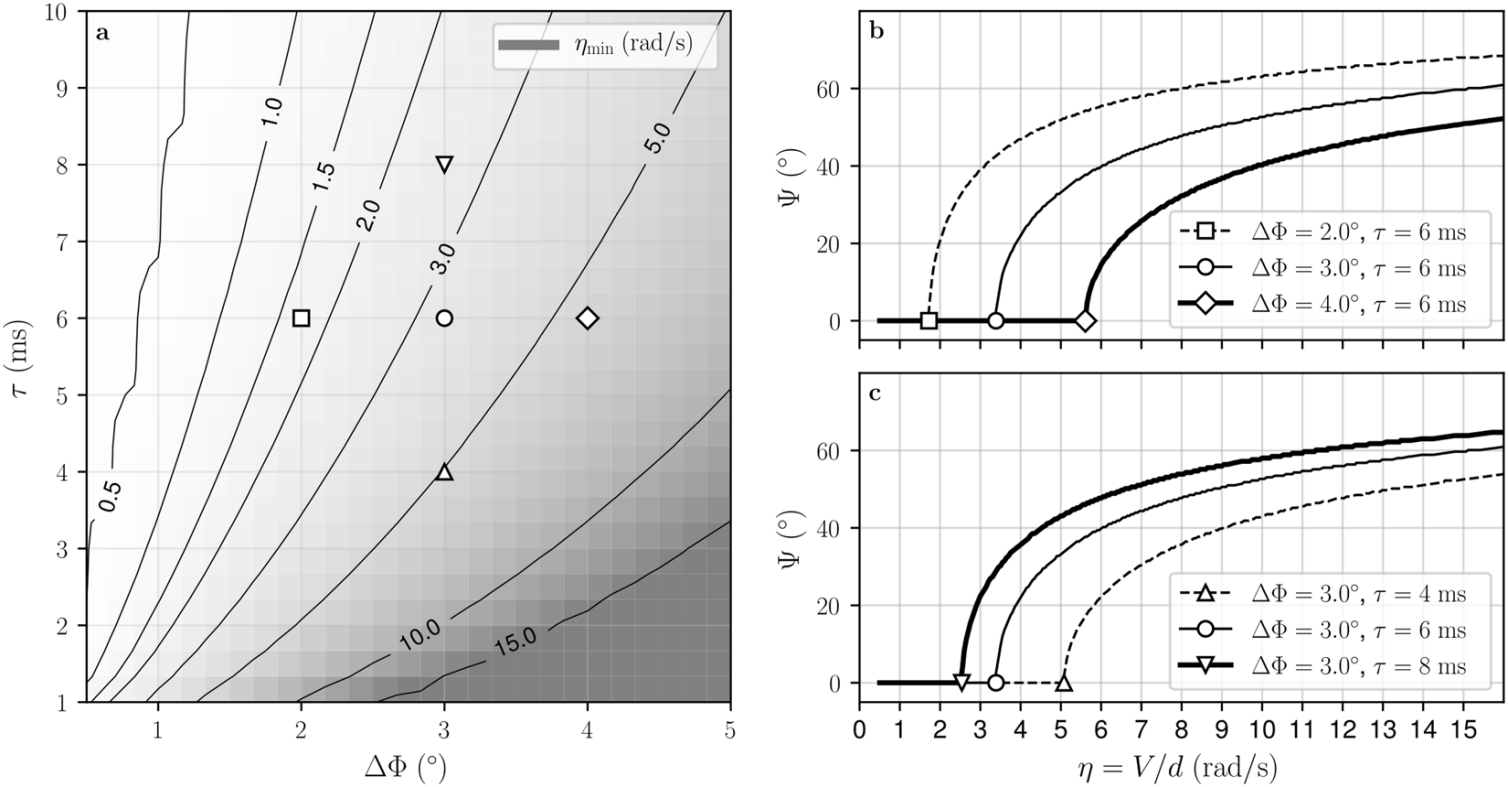
Effect of eye resolution and EMD integration time on *η*_min_ threshold and Ψ angle. (**a**) The threshold *η*_min_ is defined as the minimum *V*/*d* ratio above which Ψ can be used to estimate relative nearness (i.e. Ψ > 0°). It is presented as a function of the inter-ommatidial angle ΔΦ and the time constant *τ* of the EMD low pass filter blocks. *η*_min_ increases for increasing ΔΦ and it decreases for increasing *τ*. (**b**,**c**) The Ψ angle is presented as a function of relative nearness computed geometrically as *η* = *TOF*_90_ = *V*/*d*. Ψ is null for low relative nearness values (*η* < *η*_min_ in left portion of the graphs). When *η* > *η*_min_ (right portion of the graphs), Ψ is monotonically increasing with increasing relative nearness, and it can be used as an estimate of relative nearness. The shape of the curve $\eta \mapsto {\rm{\Psi }}$ is preserved for varying values of time constant *τ* and inter-ommatidial angle ΔΦ. (**b**) For increasing value of ΔΦ, the curve is shifted to the right, i.e. to larger relative nearness. (**c**) For increasing value of *τ*, the curve is shifted to the left, i.e. to lower relative nearness.

The threshold *η*_min_ depends on the time constant *τ* of the EMD low pass filters and on the inter-ommatidial angle ΔΦ (Fig. 4). *η*_min_ decreases with increasing time constant *τ* and increases with increasing inter-ommatidial angle ΔΦ. For example, an agent with an inter-ommatidial angle of ΔΦ = 3.0° and an EMD low pass filter constant *τ* = 10 ms will have a threshold *η*_min_ = 2 rad.s^−1^ (Fig. 4). To estimate relative nearness from Ψ (i.e. Ψ > 0°), this agent must fly at a speed of *V* > 2 m/s when it is at a distance *d* = 1 m from the surface. Similarly, it must remain at a distance of *d* < 0.5 m when flying at a speed of *V* = 1 m/s.

## Experimental Results

The proposed relative nearness estimator based on EMD is validated with closed-loop control of the lateral position and forward velocity of a simulated agent flying in a corridor with walls patterned by the surface shown in Fig. 1a. The agent can increase and decrease its forward velocity and lateral velocity. We will use the terms “forward command” and “lateral command” to refer to the velocity increments added to the forward and lateral velocity, respectively, in order to stay at equal distance to the two walls and to stabilize forward velocity at a constant value.

It is important to note that, in this section, we do not rely on the theoretical predictions of the EMD response presented in the previous section. We implemented the EMD model shown in Fig. 1b and computed its response to simulated images. The theoretical predictions only considered the steady-state EMD response to a signal with known power spectrum, while the results of this section use the actual response of the EMD model to computer-generated images.

### Control Strategy for Lateral Position and Forward Velocity

The control strategy is similar to those presented in previous studies^6,56–58^. As the agent moves forward, translational optic flow (*TOF*) is computed on its left and right sides. The difference between translational optic flow on each side is used to control the lateral position of the agent. For example, a higher translational optic flow on the right side of the agent will result in a leftward command. For speed control, the average translational optic flow on the left and right sides is compared to a reference value *TOF*_ref_. The agent will accelerate when the measured average translational optic flow is lower than the reference value, and decelerate otherwise. This control strategy can be summarised as

9 $$\begin{array}{rcl}{u}_{{\rm{lat}}} & = & {K}_{{\rm{lat}}}(TO{F}_{{\rm{left}}}-TO{F}_{{\rm{right}}})\\ {u}_{{\rm{for}}} & = & {K}_{{\rm{for}}}(TO{F}_{{\rm{ref}}}-\tfrac{(TO{F}_{{\rm{left}}}+TO{F}_{{\rm{right}}})}{2})\end{array}$$

where *u*_lat_ is the lateral command, *u*_for_ is the forward command, *K*_lat_ and *K*_for_ are proportional gains, *TOF*_left_ and *TOF*_right_ are respectively the translational optic flow measured on the left and right sides of the agent, and *TOF*_ref_ is a reference value. As the forward velocity is controlled using a reference translational optic flow value, the resulting forward velocity is expected to increase with increasing width of the corridor to compensate for the decreasing optic flow on the left and right sides.

**Figure 5:**
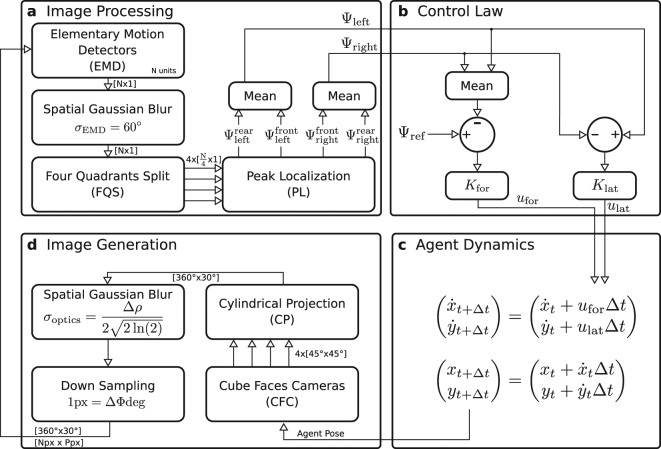
Block diagram of the simulation. (**a**) An array of N EMD units take input from consecutive pixels in the central row of a panoramic image with 360° horizontal field of view. The EMD output is spatially filtered using a gaussian kernel (with sigma *σ*_EMD_) to remove spikes and ease the detection of local maxima. Four EMD output maxima are located (PL) on each quadrant (FQS), which yields Ψ values for the rear-left, front-left, front-right and rear-right quadrants. Ψ_left_ and Ψ_right_ are obtained by taking the mean of Ψ values in the left and right hemispheres. (**b**) Forward command *u*_for_ and lateral command *u*_lat_ are computed according to equation (10). The agent is pushed towards the right (*u*_lat_ > 0) when Ψ_left_ is greater than Ψ_right_. The agent accelerates (*u*_for_ > 0) when the reference value Ψ_ref_ is greater than (Ψ_left_ + Ψ_right_)/2. (**c**) Agent dynamics are simulated as a point-mass system, where the forward velocity $\dot{x}$ and lateral velocity $\dot{y}$ are incremented using the forward and lateral commands *u*_for_ and *u*_lat_. (**d**) Four cameras (CFC) capture images that can be mapped on the faces of a cube surrounding the agent. The cameras have a field of view of 45° × 45°, are located at the agent position and are pointed at headings 0°, 90°, 180° and 270°. The cylindrical projection block (CP) converts the four cube-face images to a single image covering a field of view of 360° × 30° with all pixels on a row spanning a constant horizontal field of view. Spatial gaussian blur and sub-sampling are applied on the panoramic image to account for insect optics. The gaussian window has a sigma *σ*_optics_ defined by the acceptance angle Δ*ρ* of ommatidia. The image is down-sampled so that pixels point at directions separated by an angle equal to the inter-ommatidial angle ΔΦ. (**a–d**) In our experiments we used a simulation time-step Δ*t* = 5 ms, cube-face images with resolution 1024 × 1024 pixels and inter-ommatidial angle ΔΦ = 1°, leading to a panoramic image with a resolution of 360 × 30 pixels and *N* = 360 EMD units. The time constant of the EMD low pass filters is *τ* = 10 ms.

In our experiments, the translational optic flow values *TOF* in equation (9) are replaced with the measured Ψ values:

10 $$\begin{array}{rcl}{u}_{{\rm{lat}}} & = & {K}_{{\rm{lat}}}({{\rm{\Psi }}}_{{\rm{left}}}-{{\rm{\Psi }}}_{{\rm{right}}})\\ {u}_{{\rm{for}}} & = & {K}_{{\rm{for}}}({{\rm{\Psi }}}_{{\rm{ref}}}-\tfrac{({{\rm{\Psi }}}_{{\rm{left}}}+{{\rm{\Psi }}}_{{\rm{right}}})}{2})\end{array}$$

where Ψ_left_ and Ψ_right_ are the deviation angles of the maximum EMD response on the left and right sides, and Ψ_ref_ is a reference value (Fig. 5b).

### Simulation Environment

**Figure 6:**
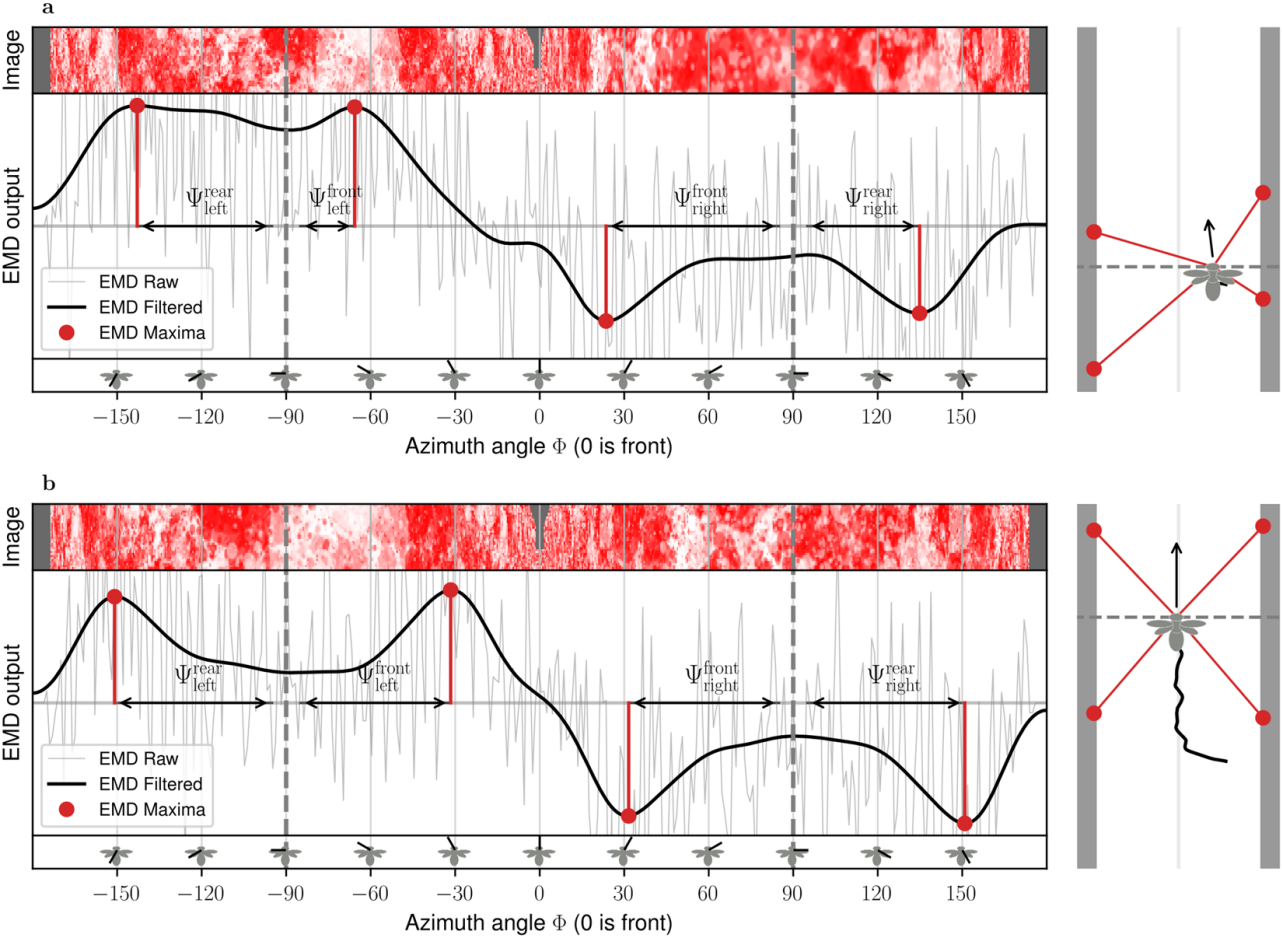
EMD Response to Simulated Images. The reponse of the array of EMDs to computer-generated images are shown at the begining of an experiment (**a**), and at the end of the experiment after the agent’s speed and position converged (**b**). The input image is shown at the top of the graph. The image is panoramic and extends from azimuth angles −180° to 180°, with the center of the image (azimuth 0°) being the front of the agent. The raw response of the array of EMDs to simulated images is represented in light grey as a function of the azimuth angle. The thick grey curve represents the signal after spatial filtering with a gaussian kernel with *σ*_EMD_ = 60°. The maximum EMD response in each of the four quadrants are shown as red dots. The deviation of the maximum EMD output in each quadrant (${{\rm{\Psi }}}_{{\rm{left}}}^{{\rm{rear}}}$, ${{\rm{\Psi }}}_{{\rm{left}}}^{{\rm{front}}}$, ${{\rm{\Psi }}}_{{\rm{right}}}^{{\rm{front}}}$ and ${{\rm{\Psi }}}_{{\rm{right}}}^{{\rm{rear}}}$) is measured between the maximum EMD output (red dots) and the side marks at azimuth −90° and +90° (grey vertical dashed lines). The drawings on the right side show the corridor seen from the top, with the position of the agent, its current speed vector and the position of the EMD maxima.

The simulated environment consists of two vertical walls covered with a “dead leaves” pattern^43,59^ (Fig. 6), which contains a naturalistic distribution of spatial frequencies. The simulation can be divided in four main steps: Image processing, Control Law, Agent Dynamics and Image Generation (Fig. 5). At each simulation time step, a new panoramic image with 360° field of view and inter-pixel angle ΔΦ is generated (Fig. 5d). The array of *N* EMD units takes input from consecutive pixels of the panoramic image, i.e. with constant inter-ommatidial angle, like our eye model (Fig. 1a). The EMD units are updated and spatially filtered, then Ψ values are computed on left and right sides from the output of the EMD units (Fig. 5a). Control commands for lateral and forward acceleration are then computed from Ψ values (Fig. 5b). Finally, the position and velocity of the simulated agent are updated based on its current state and applied control commands (Fig. 5c). The four simulation steps are repeated until the agent converges to stable flight speed and lateral position.

### Simulation Results

**Figure 7:**
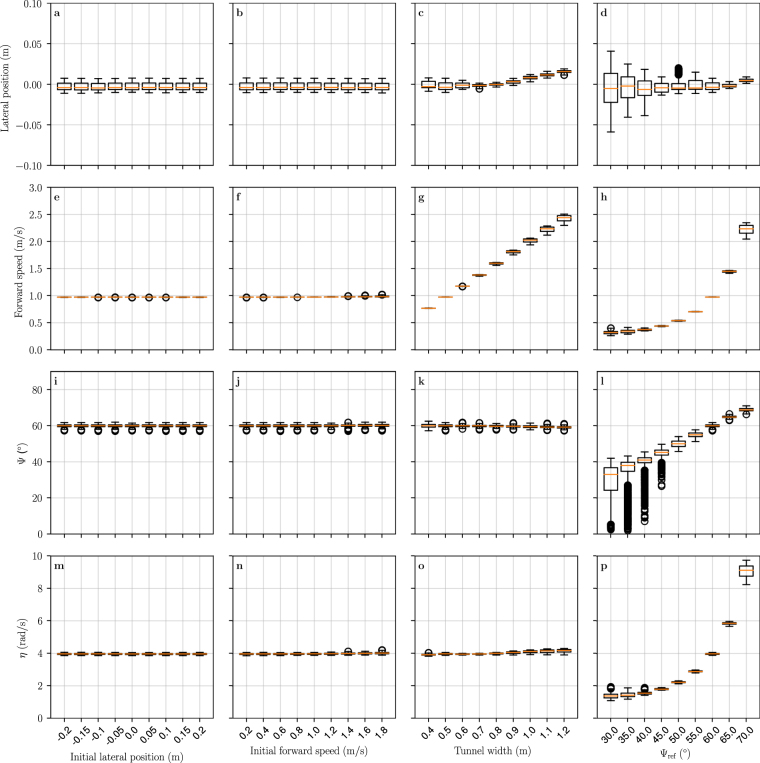
State of simulated agent after convergence. The boxplots are generated from the last 5 seconds of 10 second long flights. The first row (**a**–**d**) shows the final lateral position of the agent, 0 being the center of the corridor. The second row (**e**–**h**) shows its final forward velocity. The third row (**i**–**l**) is the measured averaged deviation of maximum EMD response (Ψ_left_ + Ψ_right_)/2. The fourth row (**m**–**p**) is the relative nearness computed as *η* = *V*/*D*, where *V* is the forward speed and *D* is the distance to the closest wall. In the first column (**a**,**e**,**i**,**m**), the initial lateral position of the agent is varied from 20 cm on the left to 20 cm on the right. The second column (**b**,**f**,**j**,**n**) shows results for varying initial forward speed. In the third column (**c**,**g**,**k**,**o**) the tunnel width is varied. In the fourth column (**d**,**h**,**l**,**p**) the commanded reference deviation of maximum EMD response Ψ_ref_ is varied. When not explicitly listed on the horizontal axis, the default initial lateral position is 0.1 m, the initial forward velocity 1 m/s, the tunnel width 0.5 m and the reference Ψ value is 60 degrees.

Simulated flights were performed with different initial lateral position, initial forward speed, tunnel width and reference command Ψ_ref_. The agent state was measured after it stabilised its velocity and lateral position (Fig. 7).

The agent converges towards the center of the corridor (lateral position equal zero) for each initial lateral position and forward velocity tested (Fig. 7a–d). The final forward speed increases with increasing tunnel width (Fig. 7g). This is an expected behaviour and matches the optic flow-based centering and speed control behaviour observed on flying insects. Indeed, this increase in speed allows the agent to maintain a constant optic flow for all tunnel widths (Fig. 7o). The Ψ angle converges to Ψ_ref_ (Fig. 7i–l), although it does so less reliably for lower Ψ_ref_ values (Fig. 7l-left). Similarly, there is higher standard deviation of the lateral position for lower Ψ_ref_ (Fig. 7d-left). The relationship between Ψ_ref_ and relative nearness (Fig. 7p) is similar to the one predicted by our analytical model (Fig. 3h). This confirms that Ψ is a correct estimate – though non-linear – of relative nearness.

An example of the EMD response during a simulation is shown in Fig. 6. At the beginning of this experiment (Fig. 6a), the agent is closer to the right wall and is flying at low speed. The Ψ angles are, on average, lower than the command Ψ_ref_ = 60°, which will push the agent to accelerate. Also Ψ angles are larger on the right side than on the left side, which will push the agent towards the left, i.e. closer to the center of the corridor. This is expected because the distance to the right wall is smaller than the distance to the left wall, so the relative nearness is higher on the right wall. Note that the raw EMD response has the inverse property: the EMD response at Φ = +90° (right) is smaller than the EMD reponse at Φ = −90° (left). Thus, if our controller had used EMD response *R*_90_ to compute the lateral command instead of Ψ, the agent would have been pushed even more to the right and would have eventually collided into the surface. At the end of this experiment (Fig. 6b), the agent is flying closer to the center of the corridor with an increased speed. The Ψ angles are all close to the command Ψ_ref_ = 60°. The agent has converged to stable lateral position and speed.

## Discussion

The EMD is a biological model for motion estimation that has received strong experimental support as the foundation of motion detection in insects. Due to its relative simplicity, the EMD model also has good potential as a computationally fast motion estimator for engineering applications. Indeed, an EMD requires two multiplications for each pixel, one subtraction and two time delays while the Lucas Kanade algorithm^60^ requires 11 multiplications and 6 subtractions. However, the EMD model output does not provide a perfect estimation of relative nearness as it cannot be unambiguously expressed in angular speed. The response of EMDs for varying angular velocity indeed has a bell shape with a maximum at an angular image velocity that is function of the EMD parameters as well as the spatial frequency of the input signal. This is problematic for biologists because insects appear to rely on relative nearness for flight control^4,5^ independently of image properties. The ambiguous nature of the EMD output is also problematic for engineers who require measures of angular speed (expressed in pixels or radians per second) for tasks such as ego-motion estimation or mapping. Also, for larger angular velocities, the EMD response decreases in a way that cannot be discriminated from a decrease in angular velocity. For example, as an agent approaches a surface – and thus as the angular image velocity increases – the response of an EMD may start decreasing. This leads to a crash into the surface when the EMD response is used to compute a repulsive force^28,29^. This case is shown in Fig. 6a where the absolute value of *R*_90_ is larger on the left side of the agent than on the right side even though the agent is offset to the right of the corridor – i.e. the angular image speed is smaller on the left side of the agent. Our simulated agent would have crashed into the wall on its right if the EMD response was used instead of Ψ to compute its lateral command.

In other words, there is an apparent incompatibility between the main neurophysiological model for motion estimation (EMD) and the main behavioural model for insect flight control (optic flow). Several studies have proposed modifications to the EMD in order to correct its output (for example^33,61–63^). Although they demonstrate improved robustness to varying contrast and spatial frequency, these models often require additional computational blocks. Most importantly, these models are less well-supported by electrophysiological recordings from the insect visual system. Here, we have shown that it is indeed possible to use a simple Hassenstein Reichardt EMD output for estimation of relative nearness with limited additional computational blocks – namely a spatial blurring and maximum location. These blocks integrate EMD responses across the visual field without modifying the structure of the correlator^13^. Because our method relies on spatial integration accross wide field of view, it is especially suited to estimation of relative nearness to a large obstacles around the agent, or to the ground below the agent.

We introduced the angle Ψ, which is the angle between the viewing direction pointing directly at the patterned surface and the viewing directions with maximal EMD response and showed that this angle is closely related to relative nearness (Fig. 3) and is therefore suitable for controlling flight (Fig. 6). Our model predicts Ψ in the limited case of straight flights parallel to a planar surface. However, we demonstrated successful flight control based on Ψ in a simulation environment that does not constrain the agent to fly along straight paths (Fig. 6-right), and also for non-planar scenes (see Supplementary Fig. S7). The main novelty of the angle Ψ is that it relies on the relative response of several EMD detectors instead of relying on the absolute value of their output. In other words, we suggest that relative nearness is spatially encoded by the relative response of EMDs rather than by the magnitude of their responses, something that has strong biological plausibility. Indeed, computing Ψ consists mostly in detecting the maximum response in an array, which is easily implemented in neural systems using a Winner-Take-All network^64^ or using differentiation and zero-crossing^9^.

While there has been much behavioural evidence that honeybees use relative nearness to control their flight^4,39^, two recent studies have shown that flight control in bumblebees does indeed exhibit some dependency on the spatio-temporal properties of sinusiodal gratings^65,66^. These apparently conflicting results can nonetheless be explained by the method we propose here because the maximum output of an array of EMDs would exhibit spatio-temporal dependencies when presented with patterns containing single frequencies (see Supplementary Figs S5 and S8) but not when presented with more complex patterns containing multiple frequencies, such as checkerboards that contain a series of discrete frequencies that are harmonics of a fundamental spatial frequency related to the size of the checkerboard squares. To avoid the ambiguities created by sinusoidal and checkerboard patterns and to make our study more relevant to the natural behaviour of insects, we considered the output of the EMD model in response to the dead-leaves pattern^43,59^, which has a spectral content that matches that of natural scenes^41,42^ with a distribution of frequency of 1/*f*  ^2^. The method we propose in this paper for extracting relative nearness from EMD output is a consequence of EMD dependency on spatial frequency, coupled with the geometry of the environment. The response of EMD is tuned to a specific ratio between image speed and angular period^33^. When insects fly above the ground or beside large flat objects, visual features of the environment are seen from a greater distance in the forward and rearward regions of their field of view. Hence, these features would subtend a smaller angle in the field of view, that is, they would appear to have a smaller angular period, i.e. a higher spatial frequency. As a consequence, the ratio of image speed to angular period at which the EMD output is maximal is achieved only at specific viewing angles, which then provides an estimate for relative nearness. Our scheme of using the angle Ψ to estimate relative nearness thus explains both the results that find spatio-temporal dependency of flight control behaviour and those that find optic flow dependency. Our scheme also highlights the importance of the structure of the pattern being used on the results of behavioural experiments on flight control.

Locating the maximum EMD response provides an estimate of relative nearness only above a threshold value *η*_min_. This means that Ψ is a valuable measure only if the agent is flying fast enough and/or close enough to the surface. Our model predicts the value of the relative nearness threshold *η*_min_ from the inter-ommatidial angle ΔΦ and the time constant *τ* of the EMD low pass filters (Fig. 4). We can investigate whether Ψ is a candidate for relative nearness information in an insect species from the speed over distance ratio *V*/*d* at which it flies and testing whether it is higher than the value of *η*_min_ that is predicted from its interommatidial angle and time constant. For an insect such as a bee, with an inter-ommatidial angle ΔΦ = 3.0° measured^50^ at an azimuth angle Φ = 90° and an estimated time constant^67^ *τ* = 10 ms, the predicted threshold is *η*_min_ = 2.0 rad.s^−1^ (Fig. 4a). This threshold is indeed lower than the flight speed to distance ratio at which bees flew in previous experiments: lateral relative nearness was recorded between 3.0 rad/s and 3.8 rad/s in *Bombus terrestris*^40,48^, and it was recorded between 3.75 rad/s and 4.96 rad/s in *Apis mellifera*^39,45^. This supports the hypothesis that these species may be using the visual angle at which maximal EMD output occurs to estimate relative nearness in order to control their flight speed. The same test can be replicated for other species using experimental measurements of ΔΦ, *τ*, and *V*/*d*.

Several studies have shown that ventral relative nearness may also be used by insects for flight control^40,48,68,69^, in addition to lateral relative nearness. Bumblebees rely primarily on lateral relative nearness cues for speed control when navigating narrow corridors, but ventral relative nearness cues are preferred over lateral relative nearness cues in wider corridors^40^. However, the lateral relative nearness in narrower tunnels (approx. 3.5 rad/s) is much smaller than the ventral relative nearness in wider tunnels (approx. 5.7 rad/s). Can this be explained by our model? Insect eyes tend to have reduced resolution in the ventral region^53^, thus, Ψ values are expected to be lower in the ventral region than in the lateral region (Fig. 4b). For the narrow corridor case (ΔΦ_lateral_ = 3.0°, *τ* = 10 ms, *η*_lateral_ = 3.5 rad/s) our model predicts Ψ_lateral_ = 40° (Fig. 4b). Within our control strategy (Fig. 5b), this corresponds to the bee maintaining Ψ equal to the reference value Ψ_ref_ = 40°. Assuming that *τ* is uniform across the eye, and that bees use the same reference Ψ_ref_ to control flight speed when using lateral motion cues and when using ventral motion cues, we can predict the ventral inter-ommatidial angle that matches the higher relative nearness in the ventral region. For the wide corridor case where *η*_ventral_ = 5.7 rad/s, Ψ_ventral_ = 40° is obtained with inter-ommatidial angle ΔΦ_ventral_ = 4.0° (Fig. 4b), which is indeed larger than ΔΦ_lateral_. In other words, with equal Ψ_ref_ and *τ* in ventral and lateral regions, but with a larger ventral inter-ommatidial angle ΔΦ_ventral_ = 4.0° than lateral inter-ommatidial angle ΔΦ_lateral_ = 3.0°, our model correctly predicts the lateral and ventral relative nearness measured in bumblebees^40^.

The relative nearness estimate provided by Ψ is most precise for *η* values slightly superior to the threshold *η*_min_. Indeed the slope of the function $\eta \mapsto {\rm{\Psi }}$ is maximum for *η* values just above the *η*_min_ threshold, i.e. a small variation in relative nearness would lead to a large variation of Ψ (Figs 3h and 4b,c). Below values of *η*_min_, however, Ψ provides no information as the slope of the function $\eta \mapsto {\rm{\Psi }}$ is null for *η* < *η*_min_ (Figs 3h and 4b,c). As a consequence, a flying agent should control its flight in order to maintain *V*/*d* values close to the threshold *η*_min_, but not below that threshold.

Conversely, adapting *η*_min_ to a value just below the currently experienced relative nearness value maximizes the precision of the Ψ estimate. Our results show that the value of the threshold can be adapted by varying the inter-ommatidial angle and the EMD time constant (Fig. 4a). A reduced inter-ommatidial angle leads to a reduced threshold (Fig. 4b) which would enable relative nearness estimates at low flight speed and/or faster reactions to obstacles that are approaching in the direction of flight. Inter-ommatidial angles are fixed by the anatomy of the compound eye and thus cannot be modified during flight. Nonetheless, the distribution of inter-ommatidial angles across the eye in different insect species may reflect adaptations that better enable Ψ estimates in relevant parts of the visual field. Another way in which the EMD output can be adapted is by modifying the time constant *τ*, which can be dynamically varied during flight^70^. An increased time constant would lead to a decreased threshold which is desirable at low flight speed, while a decreased time constant would lead to an increased threshold which is desirable at high flight speed (Fig. 4c). We suggest that a flying agent using Ψ for flight control can improve the precision of its relative nearness estimate by increasing the value of the time constant *τ* at low speed, and decreasing its value during fast forward flight. Biological evidence for dynamic changes in *τ* comes from^71^, who showed that the decreased flight speed in bumblebees that is observed in response to decreased light intensity is accompanied by an increased time constant in the photoreceptors.

The response of the EMD array to moving images contains spikes resulting from transient responses (Fig. 6 light grey). Transient EMD responses are present in our simulation but not in our model that considers only the steady-state EMD response^35^. Nonetheless, transient EMD response spikes represent measurement noise which have to be dealt with. For example, spatial differentiation and zero-crossing, which is a potential neuronal implementation for maximum detection^9^, would be strongly affected by such spikes. In simulation experiments, Ψ angles in the front and rear of the visual field were averaged (Fig. 5a), which lowers measurement noise. In addition, spatial integration of the EMD response was performed font-to-back with a spatial gaussian filter in order to remove spikes and facilitate the detection of maxima (Fig. 5a). However, the gaussian filter also flattens peaks around EMD maxima, which makes peaks difficult to disambiguate when maxima are close to each other (Fig. 6a-left), and may result in the detection of a single maxima (Ψ ≈ 0°). This is a potential explanation for the low Ψ outlier values that appear at low Ψ_ref_ angles, i.e. when EMD maxima are close to each other (Fig. 7l-left). Whether the EMD maxima can be detected and located in the presence of noise is a fundamental requirement for the applicability of our method in a real world scenario. Our model predicts the difference between the peak EMD reponse and the EMD response at Φ = 90°, i.e. the EMD response in the “well” between the two maxima (Fig. 3i–k). We showed that for low relative nearness values above the threshold *η*_min_, the EMD maxima are not only close form each other (small Ψ on the left of Fig. 3h), but also separated by a well of similar amplitude (*R*_90_ close to *R*_max_ on the left of Fig. 3k). Figure 3k shows the maximum level of measurement noise that allows the EMD maxima to be distinguished from the EMD response at 90 degrees. To obtain a reliable estimate of relative nearness using the EMD output, it is not only necessary to keep the *V*/*d* value above the *η*_min_ threshold, but it is also necessary to keep a margin above *η*_min_ in order to have clearly separated peaks in the EMD response (Fig. 6b). Biological data discussed previously^39,40,45,48^ suggest that bees fly with Ψ_ref_ = 40°, which means that EMD maxima would be separated by a comfortable angle of 80°.

How does our method for estimation of relative nearness generalize to a two dimensional field of view? With a 2D spherical field of view, a viewing direction is defined by its elevation angle Θ in addition to the azimuth angle Φ. The present study assumes an elevation angle equal to zero. In equation (5), the apparent temporal frequency $\widehat{\omega }$ is not be affected by varying elevation angle, the same way it is not affected by varying azimuth angle. The geometry of the environment is axially symmetric about the viewing direction with azimuth Φ = 90° and elevation Θ = 0° (Fig. 1a) and so is the apparent angular period $\widehat{\lambda }$ and apparent signal amplitude $\widehat{{\rm{\Delta }}I}$. As a consequence, the EMD output *R* is constant over circles centered on the viewing direction $$[\begin{array}{c}{\rm{\Phi }}\\ {\rm{\Theta }}\end{array}]=[\begin{array}{c}90\\ 0\end{array}]$$. In other words, with a 2D EMD array, there are not only two maxima in the EMD response. Instead, there is a circle where the EMD response is maximal. This circle is centered on viewing direction $$[\begin{array}{c}{\rm{\Phi }}\\ {\rm{\Theta }}\end{array}]=[\begin{array}{c}90\\ 0\end{array}]$$, and the radius Ψ of the circle can be used as an estimate of realtive nearness. Note that this circle contains the two viewing directions with maximum EMD response presented in this study $$([\begin{array}{c}{\rm{\Phi }}\\ {\rm{\Theta }}\end{array}]=[\begin{array}{c}90-{\rm{\Psi }}\\ 0\end{array}]\,{\rm{and}}\,[\begin{array}{c}{\rm{\Phi }}\\ {\rm{\Theta }}\end{array}]=[\begin{array}{c}90+{\rm{\Psi }}\\ 0\end{array}])$$ and is thus an extension of the 1D case. The immediate benefit of using two dimensional field of view is the reduction of measurement noise by spatial integration. Instead of performing spatial integration front-to-back (Fig. 5a), it could be performed using the second dimension of the image — along circular paths — in order to measure the average radius Ψ of the circle where EMD response is maximum. We expect that with this method, maxima would be easier to distinguish even at low Ψ angles, while still filtering transient EMD response spikes. Whether this two dimensional spatial integration is used by insects could be tested by checking whether the output of motion detecting neurons are spatially pooled across regions with circular shape.

## Conclusion

In this paper, we modeled the response of an array of EMDs in the case of an agent flying along a flat patterned surface and showed that the raw value of the EMD response is poorly correlated to relative nearness. We showed that the location of the maximum response in the EMD array provides appropriate estimation of relative nearness when the agent is flying sufficiently fast and/or close to the surface. We introduced the notion of relative nearness threshold to provide bounds on speed and distance, and showed that they are consistent with data from flight control experiments on *Bombus terrestris* and *Apis mellifera*. Finally, we proposed a flight control strategy that uses the location of maximum EMD response as control input instead of optic flow and tested it in a 3D simulation where we successfully controlled the forward velocity and lateral position of a simulated agent flying in a corridor. Similar to what is observed in insects, and as expected with optic flow based control, the agent’s forward velocity is dependent on the corridor width: the broader the corridor, the faster the agent advances.

The method of extracting relative nearness from EMD output that is described here relies on a standard form^14^ and requires few additional computational steps: namely, spatial filtering and detection of maximum EMD response, with both being easily modeled as neuronal networks. Further studies are needed in order to investigate if this scheme is indeed used in biological systems and to identify its neural underpinnings. Nevertheless, our method provides an algorithm for estimation of relative nearness that has low computational cost and that could be readily used in robotics applications.

### Data availability

The datasets generated during and/or analysed during the current study are available from the corresponding author.

## Electronic supplementary material

Supplementary Material
