# Supplementary material for: Spatial Encoding of Translational Optic Flow in Planar Scenes by Elementary Motion Detector Arrays
Source: Sci Rep. 2018 Apr 11;8:5821. doi: 10.1038/s41598-018-24162-z (PMC5895815; doi:10.1038/s41598-018-24162-z)
Supplement: Supplementary file 1 — Supplementary Material [file 41598_2018_24162_MOESM1_ESM.pdf]

Spatial Encoding of Translational Optic Flow in Planar Scenes by Elementary Motion Detector Arrays

Julien Lecoeur[1,*], Emily Baird[2,+], and Dario Floreano[1,+]

[1]Laboratory of Intelligent Systems, School of Engineering, École Polytechnique Fédérale de Lausanne, Lausanne, CH-1015, Switzerland
[2]Department of Biology, Lund University, Lund, Sweden
[*]julien.lecoeur@epfl.ch
[+]these authors contributed equally to this work

S1 Values of parameters for theoretical study

The model was evaluated with $N_f = 2000$, $f_{min} = 1$, and $f_{max} = 1000$. The five parameters τ, $\Delta\Phi$, d, V, and Φ were linearly sampled according to the values presented in the following table.

	minimum	maximum	Number of values
f	1 m^{-1}	1000 m^{-1}	2000
τ	1 ms	10 ms	28
$\Delta\Phi$	0.5°	5°	28
d	0.05 m	0.2 m	49
V	0.1 m/s	0.8 m/s	49
Φ	$\pi/24$ rad	$\pi/2$ rad	200

The speed of the agent V was sampled with 49 different values between 0.1m/s and 0.8m/s. The distance d between the agent and the surface was sampled with 49 different values between 5cm and 20cm. The inter-ommatidial angle $\Delta\Phi$ was sampled with 28 different values between 0.5° and 5°. The time constant τ was sampled with 28 different values between 1 ms and 10 ms The azimuth angle Φ was sampled with 200 different values between $\pi/24$ and $\pi/2$.

The table below presents the values of the apparent angular period measured at $\Phi = 90°$ for extreme values of linear frequency f and distances d.

λ_{90} (°)	d_{min}	d_{max}
f_{min}	168.58	136.40
f_{max}	1.15	0.29

The table below presents the values of temporal frequencies for extreme values of linear frequency f and speeds V.

$\nu = \frac{\omega}{2\pi}$ (cycles/s)	V_{min}	V_{max}
f_{min}	0.1	0.8
f_{max}	100.0	800.0

S2 Expressions of apparent angular period, apparent temporal and linear frequency, and apparent signal amplitude

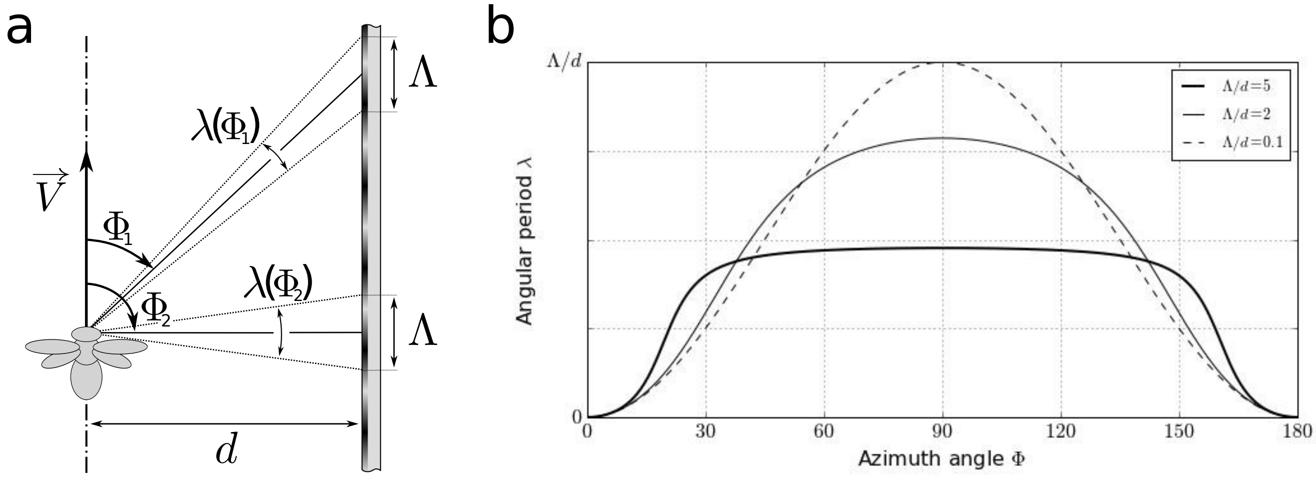

Figure S2. Apparent angular period for varying viewing direction Φ and ratio between Λ and d. For $\Lambda << d$, the small angle approximation holds true and the apparent angular period follows a squared sinus as is expected in the case of a lateral infinite wall. However when $\Lambda > d$, the apparent angular period is shorter on the side ($\Phi \approx 90°$), and longer in front and in the back ($\Phi \rightarrow 0°$ and $\Phi \rightarrow 180°$).
.

Apparent angular period

The apparent angular period $\widehat{\lambda}$ is defined, for a given linear period Λ, as the angular size occupied by a complete cycle on the retina of the agent as shown on Fig. S2a. It is dependent on the distance to the surface d, the linear period Λ and the viewing direction Φ. By posing $x^+ = d\tan(\theta) + \frac{\Lambda}{2}$, $x^- = d\tan(\theta) - \frac{\Lambda}{2}$, and $\theta = \frac{\pi}{2} - \Phi$, we obtain geometrically from Fig. S2:

$$\widehat{\lambda} = \arctan\left(\frac{x^+}{d}\right) - \arctan\left(\frac{x^-}{d}\right) \tag{S1}$$

Thus the expression for the apparent angular period is given by equation (S2). The variation of the apparent angular period across the visual field can be seen on Fig. S2b.

$$\widehat{\lambda} = \arctan(\tan(\frac{\pi}{2} - \Phi) + \frac{\Lambda}{2d}) - \arctan(\tan(\frac{\pi}{2} - \Phi) - \frac{\Lambda}{2d}) \tag{S2}$$

Apparent temporal and linear frequency

Linear frequencies, linear periods and angular periods are respectively noted f, Λ and λ, and are respectively expressed in m^{-1}, m, and radians. The relation between f and Λ is given by equation (S3).

$$f = \frac{1}{\Lambda} \tag{S3}$$

Temporal frequency ω is expressed in radians per second. In the case presented in Fig. S2a, the temporal frequency does not depend on the viewing direction Φ and is only function of the speed of the agent and the linear period as given in equation (S4).

$$\widehat{\omega} = 2\pi\frac{V}{\Lambda} \tag{S4}$$

Apparent signal amplitude

In a viewing direction Φ, a sinusoidal pattern is seen with an apparent angular period λ through photoreceptors with Gaussian acceptance windows of standard deviation $\sigma = \frac{\Delta\rho}{2\sqrt{2\ln(2)}}$. The apparent signal amplitude is the result of the convolution of the input signal with this Gaussian window as in equation (S5).

$$\widehat{\Delta I} = \exp\left(-2\pi^2 \frac{\sigma^2}{\lambda^2}\right) \tag{S5}$$

According to[1], in most diurnal insect species the ratio between acceptance angle and inter-ommatidial angle is given by equation (S6):

$$\frac{\Delta\rho}{\Delta\Phi} \approx 1.07 \tag{S6}$$

From equation (S5) and equation (S6), we can express the apparent signal amplitude as a function of the inter-ommatidial angle and the apparent angular period, as given in equation (S7).

$$\widehat{\Delta I} \approx \exp\left(-2\pi^2 \frac{(0.45\ \Delta\Phi)^2}{\lambda^2}\right) \tag{S7}$$

S3 EMD velocity response curve for a broadband signal

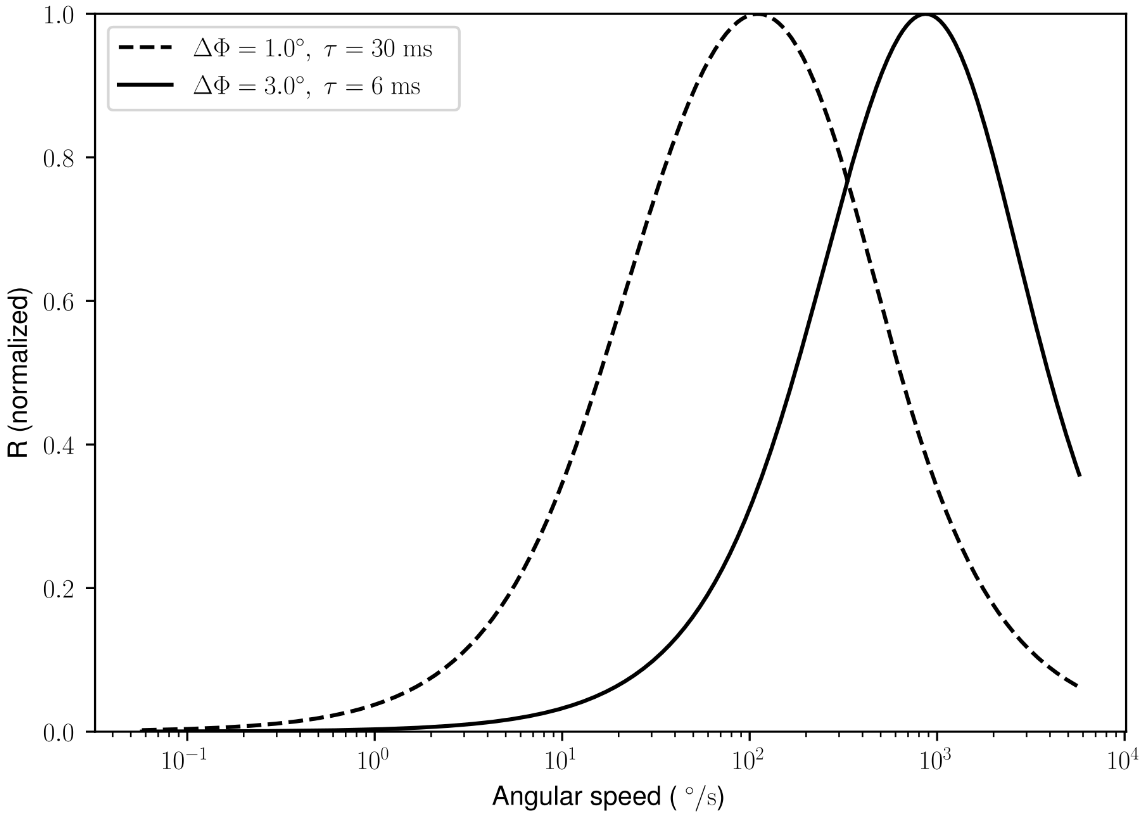

Figure S3. EMD velocity response curve for a broadband signal. The input signal has a $1/f^2$ distribution of spatial frequencies. The time constant and the interommatidial angle of the EMD are set respectively to $\tau = 6$ ms and $\Delta\Phi = 3°$ (solid curve), and $\tau = 30$ ms and $\Delta\Phi = 1°$ (dashed curve). The data is computed at viewing angle $\Phi = 90°$ by varying flight speed V at fixed distance $d = 0.1$ m from the pattern considered in the paper.

S4 EMD velocity response curve for sinusoidal signals

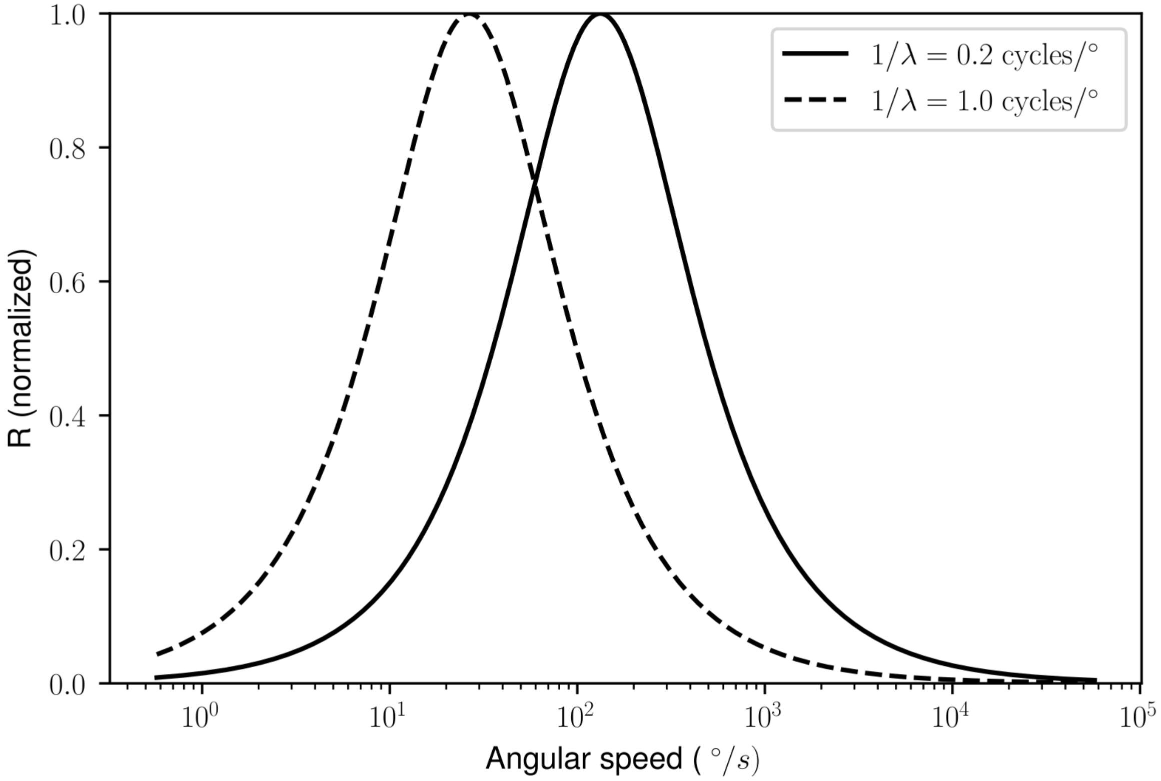

Figure S4. **EMD velocity response curve for sinusoidal signals.** The sinusoidal signals have apparent angular frequencies of $1/\lambda = 0.2$ cycles/° and $1/\lambda = 1.0$ cycles/°. The time constant of the EMD is set to $\tau = 6$ ms and the interommatidial angle to $\Delta\Phi = 3°$. The data is computed by varying flight speed V at fixed distance $d = 0.1$ m from sinusoidal gratings with spatial period Λ chosen in order to obtain the desired apparent angular periods λ at viewing angle $\Phi = 90°$.

S5 EMD response and Ψ angle for single-frequency pattern

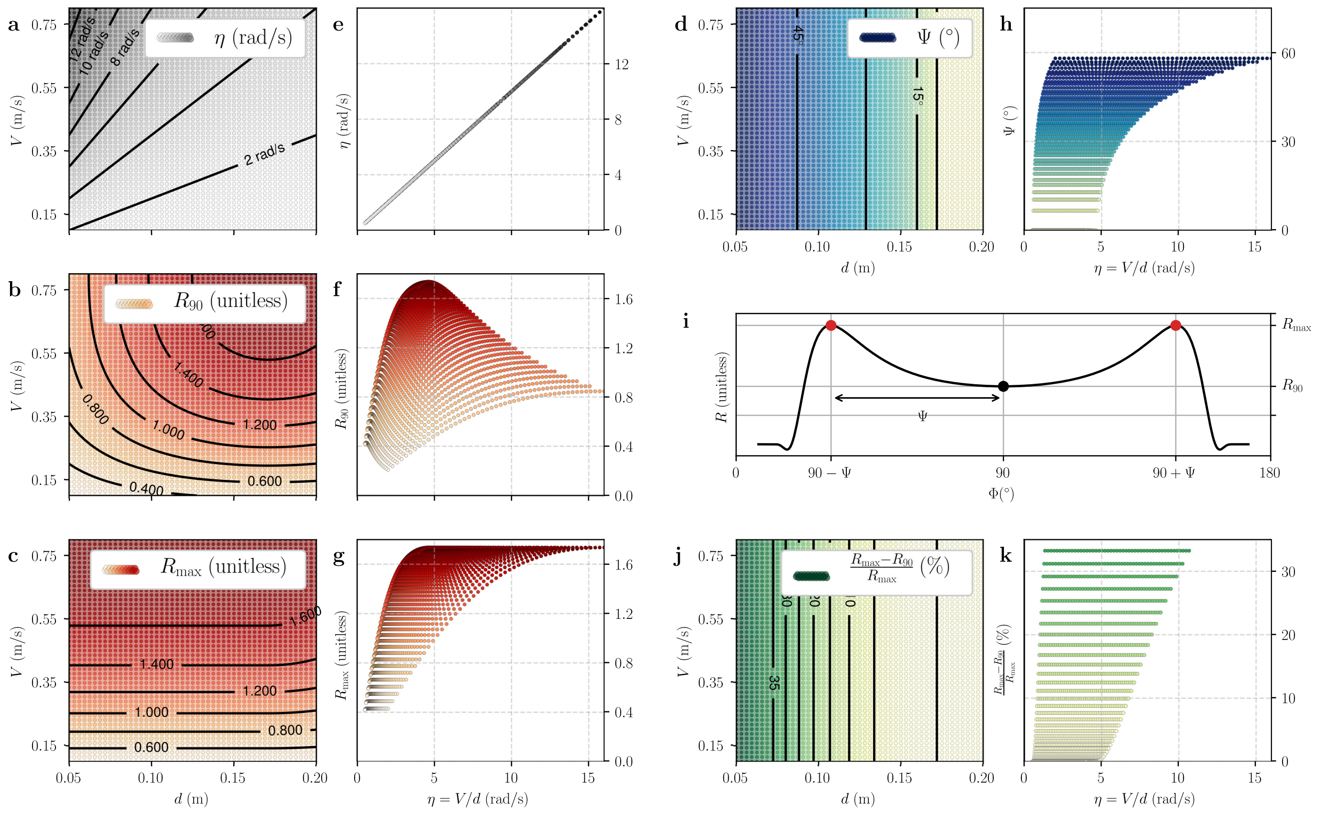

Figure S5. **EMD response and Ψ angle for single-frequency pattern.** The data shown here should be compared to that shown in the main text (Fig. 3, titled "Comparison of raw EMD response and Ψ angle as estimators of relative nearness"). The main text considers broadband images consisting as a sum of sinusoidal components with spatial frequencies in the range $[f_{\min}, f_{\max}]$. Here we reproduced the same study for a single-frequency image, i.e. a sinusoidal grating. The spatial period of the sinusoidal grating is $\Lambda = 5$ cm, which is in the range of spatial periods used in the main text. We can see that Ψ no longer encodes relative nearness (**h**). Also it is noteable that contrary to the broadband case, $R_{\max}$ is mostly independent from the distance (**c**), and Ψ is mostly independent from the flight speed (**d**). (**a**) and (**e**): Relative nearness, computed geometrically as $\eta = V/d$. The unit is rad/s because this is equivalent to the angular image speed. (**b**) and (**f**): The EMD response at 90° is defined as $R_{90} = R_{(\Phi=90°)}$. (**c**) and (**g**): The maximum EMD response is defined as $R_{\max} = R_{(\Phi=\Phi_{\max})}$. (**d**) and (**h**): Deviation of the location of maximum EMD response $\Psi = |\Phi_{\max} - 90°|$. Left (**a-d**): Values given as functions of flight speed V and distance d. Right (**e-h**): Values given as functions of the relative nearness η which is equivalent to the translational optic flow at viewing angle 90 degrees $\eta = TOF_{90} = V/d$. In all plots, the inter-ommatidial angle and time constant of the low pass filter are kept constant at $\Delta\Phi = 3°$ and $\tau = 10$ ms. (**i**): Graphical representation of R_{90}, $R_{\max}$ and Ψ on EMD response R shown as function of viewing angle Φ. (**j-k**): Relative difference between $R_{\max}$ and R_{90} (given in percents), it indicates the maximum level of noise allowing the two maxima to be detected.

S6 Simulation environment

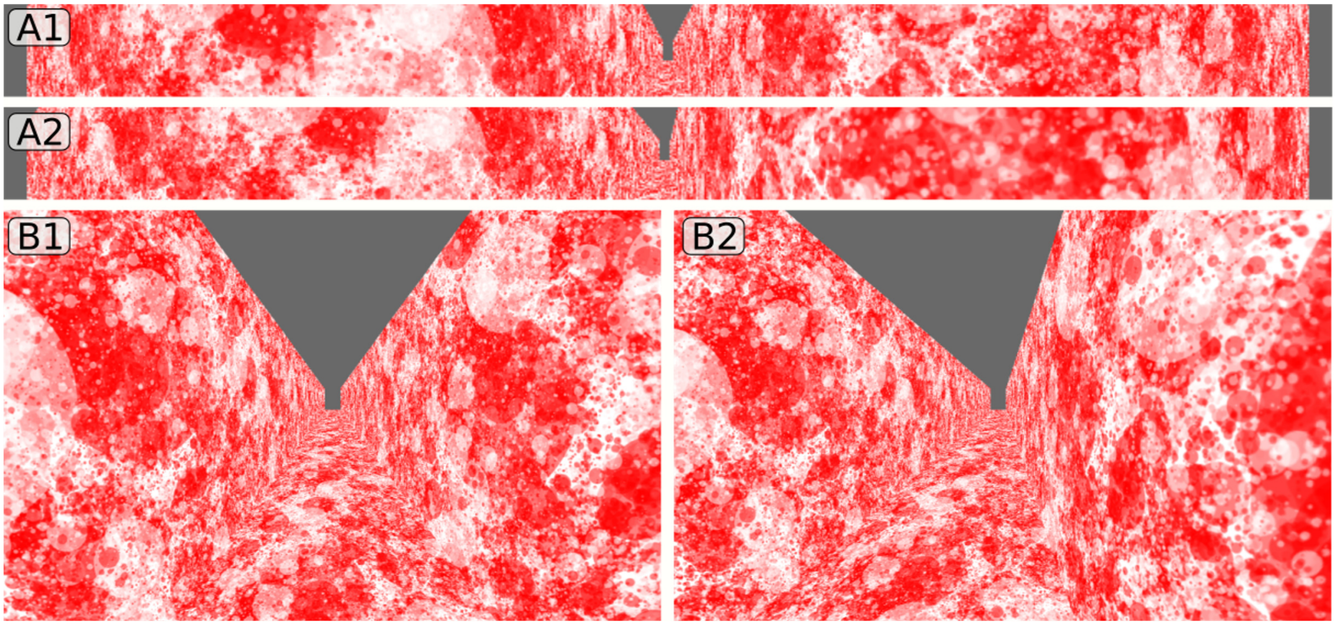

Figure S6. Simulation environment. A1 and A2: Images generated by a camera with cylindrical projection used by our simulated agent. The cylindrical projection camera has a lateral field of view of 360 degrees and vertical field of view of 30 degrees. A1 is an view from the center of the corridor. A2 is a view with the agent flying closer to the right wall. B1 and B2: Images generated using a forward looking fish eye camera used for reference only. The simulated agent does not use these images. The image B1 is taken from the same location as the image A1, similarly the image B2 is taken from the same location as the image A2.

S7 Simulated trajectories in non-planar scenes

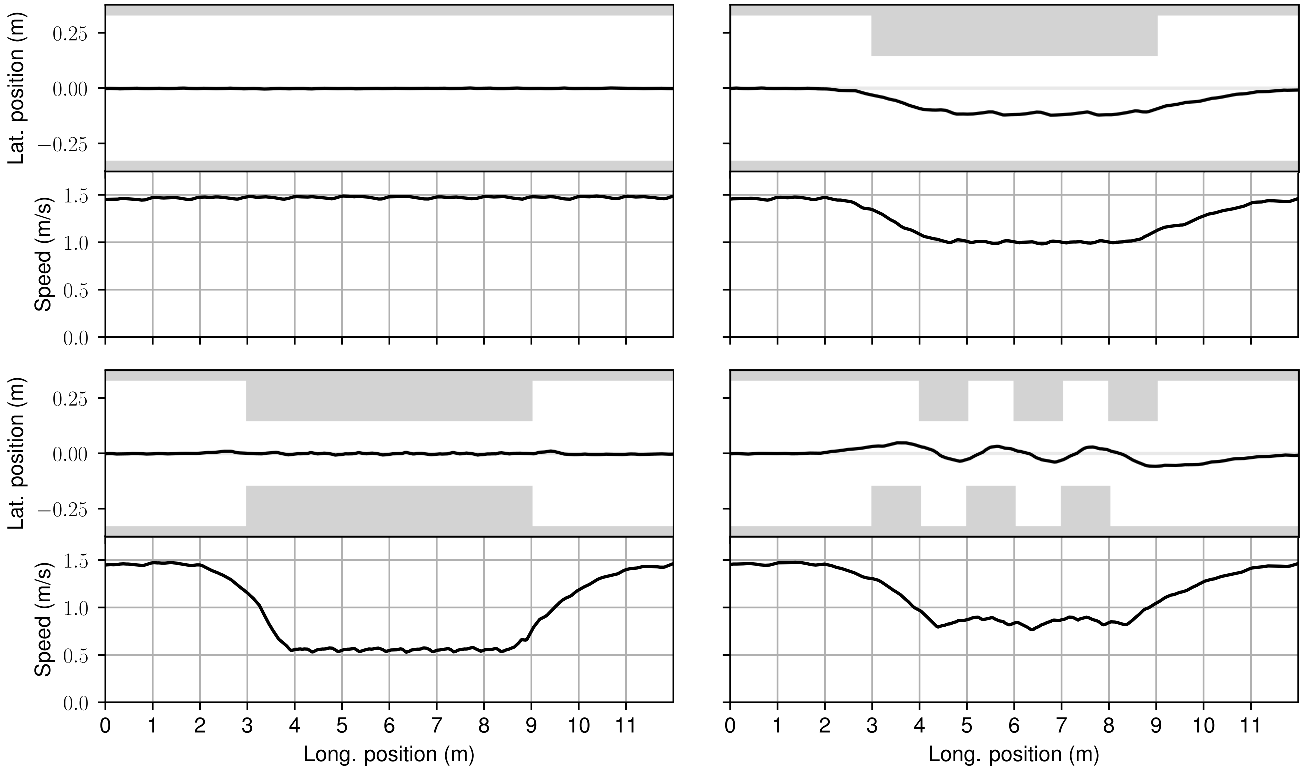

Figure S7. Simulated trajectories in non-planar scenes. Our theoretical model is derived for the case of straight flights parallel to a flat surface, hence it cannot predict Ψ values in non-planar scenes. However the robustness of our proposed relative nearness estimation technique and associated control method can be tested in more realistic scenarios. To this effect, we run the same simulation as in section ”Experimental results” of the main text, with the addition of obstacles on the path of the agent. The obstacles occupy 30% of the corridor width. The initial lateral position is 0.0 m, the initial forward velocity 1.5 m/s, the corridor width is 0.75 m and the reference Ψ_{ref} value is 60 degrees. (**Top left**): No obstacle. This is the case presented in the main text. The agent flies at constant speed of 1.5 m/s in the center of the corridor. (**Bottom left**): Two obstacles are placed on both sides of the corridor. The agents stays in the center of the corridor. Approximately 1 meter before reaching the obstacles (i.e at longitudinal position of 2 meters), the agent starts to slow down. Its speed then stabilises at a value slighlty above 0.5 m/s, which is coherent with the 60% reduction in corridor width. Approximately 0.5 meter before reaching the end of the narrow section, the agent starts to increase its speed until it recovers its original speed. (**Top right**): One obstacle is placed on the left side of the corridor. As it approches the obstacle, the agent slows down and moves towards the right the corridor in order to regulate Ψ values on left and right sides to Ψ_{ref}. (**Bottom right**): Six obstacles are placed alternatively on the left and right sides of the corridor in order to form a slalom. As expected the agent slows down when aproaching the obstacles, and it steers left and right in order to stay away from the obstacles and avoid collisions.

S8 Simulated trajectory with single-frequency pattern

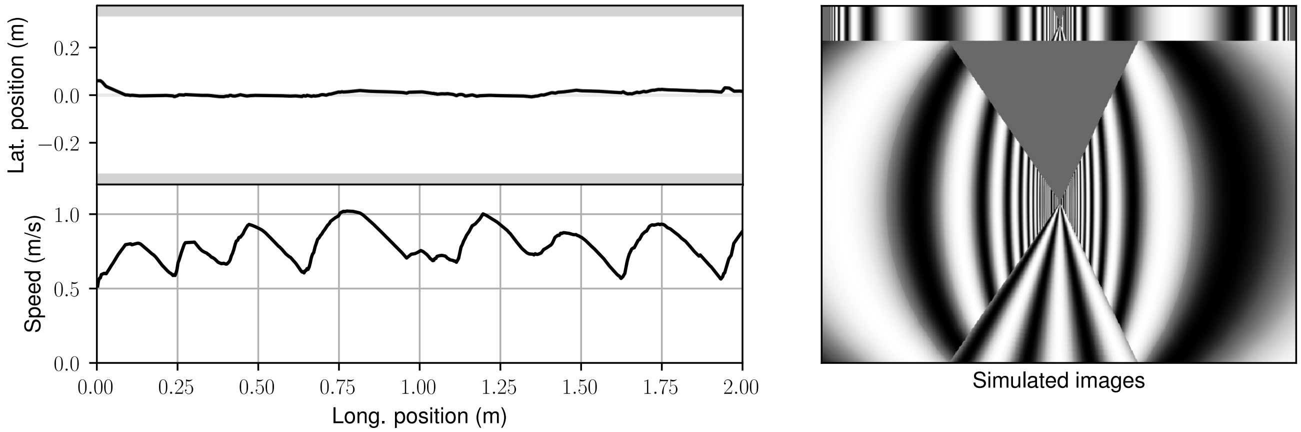

Figure S8. **Simulated trajectory with single-frequency pattern** The data shown here complements the theoretical predictions made in Supplementary Figure S5. The main text considers broadband images consisting as a sum of sinusoidal components with spatial frequencies in the range $[f_{\min}, f_{\max}]$. Here we reproduced the experiment presented in the section ”Experimental Results” of the main text, but replaced the broadband images by a single-frequency image, i.e. a sinusoidal grating. The initial lateral position is 0.1 m, the initial forward velocity 0.5 m/s, the corridor width is 0.4 m and the reference Ψ_{ref} value is 60 degrees. (**Right**): The walls of the corridor are covered with a sinusoidal grating with linear period $\Lambda = 33$ cm. (**Top left**): In the single-frequency case, our proposed control method allows the agent to stabilise its lateral position in the center of the corridor. This is expected from the prediction (showed in Supplementary Figure S5) that Ψ decreases with the distance to the walls. (**Bottom left**): In the single-frequency case, the agent is not able to stabilise its flight speed, it varies erratically between 0.5 m/s and 1.0 m/s. This is expected from the prediction (showed in Supplementary Figure S5) that Ψ is independent from the flight speed. It should be noted that a natural setting would realistically not include single-frequency patterns. Furthermore insects are likely to control flight speed using additional regions of the visual field (like the ventral and dorsal regions) that may contain patterns with richer frequency content.

References

1. Land, M. F. Visual Acuity in Insects. *Annual Review of Entomology* **42**, 147–177 (1997).
